# Localization of Calretinin, Parvalbumin, and S100 Protein in *Nothobranchius guentheri* Retina: A Suitable Model for the Retina Aging

**DOI:** 10.3390/life13102050

**Published:** 2023-10-13

**Authors:** Marialuisa Aragona, Marilena Briglia, Caterina Porcino, Kamel Mhalhel, Marzio Cometa, Patrizia Germana Germanà, Giuseppe Montalbano, Maria Levanti, Rosaria Laurà, Francesco Abbate, Antonino Germanà, Maria Cristina Guerrera

**Affiliations:** Zebrafish Neuromorphology Lab, Department of Veterinary Sciences, University of Messina, 98168 Messina, Italy; mlaragona@unime.it (M.A.); marilena.briglia@unime.it (M.B.); kamel.mhalhel@unime.it (K.M.); marzio.cometa@unime.it (M.C.); pgermana@unime.it (P.G.G.); gmontalbano@unime.it (G.M.); mblevanti@unime.it (M.L.); laurar@unime.it (R.L.); abbatef@unime.it (F.A.); agermana@unime.it (A.G.); mariacristina.guerrera@unime.it (M.C.G.)

**Keywords:** CaBPs, retina, aging, *N. guentheri*

## Abstract

Calcium-binding proteins (CaBPs) are members of a heterogeneous family of proteins able to buffer intracellular Ca^2+^ ion concentration. CaBPs are expressed in the central and peripheral nervous system, including a subpopulation of retinal neurons. Since neurons expressing different CaBPs show different susceptibility to degeneration, it could be hypothesized that they are not just markers of different neuronal subpopulations, but that they might be crucial in survival. CaBPs’ ability to buffer Ca^2+^ cytoplasmatic concentration makes them able to defend against a toxic increase in intracellular calcium that can lead to neurodegenerative processes, including those related to aging. An emergent model for aging studies is the annual killifish belonging to the Nothobranchius genus, thanks to its short lifespan. Members of this genus, such as *Nothobranchius guentheri*, show a retinal stratigraphy similar to that of other actinopterygian fishes and humans. However, according to our knowledge, CaBPs’ occurrence and distribution in the retina of *N. guentheri* have never been investigated before. Therefore, the present study aimed to localize Calretinin N-18, Parvalbumin, and S100 protein (S100p) in the *N. guentheri* retina with immunohistochemistry methods. The results of the present investigation demonstrate for the first time the occurrence of Calretinin N-18, Parvalbumin, and S100p in *N. guentheri* retina and, consequently, the potential key role of these CaBPs in the biology of the retinal cells. Hence, the suitability of *N. guentheri* as a model to study the changes in CaBPs’ expression patterns during neurodegenerative processes affecting the retina related both to disease and aging can be assumed.

## 1. Introduction

Ca^2+^ ions can regulate a wide range of intracellular mechanisms. For instance, at the synaptic level, the transmission of a chemical stimulus in neurons depends on calcium concentration. When a transmembrane flow of Ca^2+^ ions is generated, a local increase in the intracellular concentration of the ion at the presynaptic termination regulates the fusion of vesicles containing neurotransmitters with the plasma membrane [1,2,3,4] and the release of their contents into the synaptic fissure [3,5,6]. Intracellular Ca^2+^ mobilization is involved in several downstream mechanisms induced by the activation of the GPCRs that evoke slow synaptic transmission [7,8]. The calcium level within cells is regulated by many proteins such as channels, transporters, pumps, and CaBPs. Calcium efficacy as a signaling agent depends on the level of sequestration of cytoplasmic calcium. In particular, some CaBPs are proteins that can act like buffers [9] establishing gradients of free Ca^2+^ near a Ca^2+^ source, like a membrane ion channel [10,11]. CaBPs’ ability to bind Ca^2+^ is relevant in the context of aging and neurodegeneration, because according to the so-called “Ca^2+^ aging hypothesis”, intracellular Ca^2+^-concentration is involved in age-related neurodegeneration [12,13]. Distinct studies showed the link between senescence decline and progressive increases in Ca^2+^ influx in cerebral cells [14,15,16]. In this context, the action of the calcium protein family ligands (CaBPs) can act as neuroprotective factors containing age-related changes. Calcium binding proteins are involved in calcium signaling regulation and neuronal excitability modulation for the long-term [17]. Calretinin, parvalbumin, and the S-100 protein are often considered histological markers due to their distribution in neuronal subclasses [14,18]. CaBPs are extensively disseminated in the peripheral and central nervous system, including the retina [14,15,16]. Since the retina is a crucial component of the central nervous system (CNS), disease processes in the retina could indicate a similar process elsewhere in the CNS and vice versa. In addition, scientific evidence [19,20,21,22] has shown that retinopathies appear concurrently with neurodegenerative diseases.

The brain and retina share this common aging mechanism. Even if retina aging and its related neurodegenerative process are complex due to the combination of multiple genes, cell processes and death, biochemical alterations, and environmental risk factors [14,15,16,17,18] to study the role of Calcium and CaBPs are intriguing. Indeed, it has been demonstrated that during retinal aging, a higher level of intracellular calcium promotes the weakening of synapses and the degradation of visual performance, as demonstrated by Berkowitz et al. [23]. Likewise, age-related changes in calcium-binding proteins and its different trends in the SNC and in the retina have been reported in several species [18,24,25,26,27,28,29,30,31,32,33,34,35,36,37,38,39]. Different CaBPs, like calretinin and parvalbumin, can be used as cellular markers in retinal aging studies to monitor these changes over a lifetime [40]. It has been demonstrated that vertebrates from fish to mammals express CaBPs both in the central and peripheral nervous systems [4,5,6,7,41,42,43,44], and it is intriguing in the translational medicine field. Aging studies have been impaired because of the lack of a suitable model. However, *Nothobranchius* spp. is being established as a suitable model in this research area. For instance, *Nothobranchius guentheri* (*N. guentheri*) retina shows the typical retinal stratigraphy of vertebrates including humans: ganglion cell layer (GCL), inner plexiform layer (IPL), inner nuclear layer (INL), outer plexiform layer (OPL), outer nuclear layer (ONL), inner segment/outer segment of photoreceptor cells (PRL), retinal pigment epithelium cells (RPE) [45]. The Nothobranchiidae family of fish is a large group, typical of North Africa where they mainly inhabit shallow ephemeral pools and seasonal swamps. The *Nothobranchius* spp. hold the record of the fastest maturing vertebrate with the briefest life in captivity and is a novel model organism in aging studies [46]. Further, *N. guentheri* and humans share some aging markers, for instance, the apolipoprotein E [26], the insulin growth factor [16,26,27], and isthmin [28]. It has also been demonstrated that the expression of senescence-associated β-galactosidase and the accumulation of lipofuscin increased with age both in Nothobranchius and humans [29,30]. In the aging retina of both in Nothobranchius and humans, a decreased effectiveness of the antioxidant defense system occurs [30,31], so the activities of catalase, glutathione peroxidase, and superoxide dismutase decreased with age. For all the abovementioned reasons, this work aimed to show the localization of calcium-binding proteins in the retina of the annual killifish (*N. guentheri*) as a possible model for retinal aging studies.

## 2. Materials and Methods

### 2.1. Fish and Tissue Treatment

In this investigation, we used paraffin-embedded tissue of 1-year-old *N. guentheri* from earlier studies [32]. One-year-old, male, and female adult *N. guentheri* specimens from ornamental aquariums were employed. They were found dead of unexplained causes. The heads were rapidly removed and stored in 4% paraformaldehyde (Sigma-Aldrich, Inc., St. Louis, MO, USA, #158127) in 0.1 m (pH = 7.4) of phosphate-buffered saline (PBS, Sigma-Aldrich, Inc., St. Louis, MO, USA, # P4417) for 12–18 h, dehydrated by graded ethanol series, clarified in xylene, and used for paraffin wax (Bio-Optica S.p.a. Milano, Italy, # 08–7910) embedding. The included tissues were then cut into serial sections that were 7 μm thick and collected on gelatin-coated microscope slides [47]. Later, deparaffinized and rehydrated serial slices were washed in distilled water, processed with Masson trichrome with aniline blue (Bio-Optica S.p.a Milano, Italy, cat. #04-010802) [33]. At the end, stained sections were examined under a Leica DMRB light microscope equipped with Leica MC 120 HD camera (Leica Application Suite LAS V4.7).

### 2.2. Immunohistochemistry

To analyze the localization of CaBPs in *N. guentheri* retina, some serial slides were deparaffinized and rehydrated, finally in phosphate-buffered saline (PBS Sigma-Aldrich, Inc., St. Louis, MO, USA cat. # P4417). The sections were incubated in 0.1% Triton X100 (Sigma-Aldrich, Inc., St. Louis, MO, USA, cat. #X100) PBS solution to permeate the membranes, after incubation in a 0.3% hydrogen peroxide solution (H_2_O_2_ Sigma-Aldrich, Inc., St. Louis, MO, USA, cat. #1085971000) to prevent the activity of endogenous peroxidase. The 25% fetal bovine serum (Sigma-Aldrich, Inc., St. Louis, MO, USA, cat. #F7524) solution was then added to the rinsed sections. Sections were incubated overnight at 4 °C in a humid chamber with antibodies ant-Calretinin N-18 and anti-Parvalbumin antibodies (see Table 1). Some representative sections were incubated with anti-Opsin and anti-Chat antibodies, recognized as specific markers for rods and amacrine cells, respectively (see Table 1). Some representative serial sections were used to detect anti-S100 protein that recognizes a mixture of both S100A and S100B proteins subunit by the immunohistochemistry peroxidase method. As mentioned above, sections were incubated overnight with anti-S100 protein (see Table 1). After rinsing in PBS, the sections were incubated for 1 h at 4 °C with a fluorescent secondary antibody (see Table 1) at room temperature in a dark humid chamber. Washing and mounting using Fluoromount Aqueous Mounting Medium (Sigma-Aldrich, Inc., St. Louis, MO, USA, cat. #F4680) were the final steps. A Zeiss LSMDUO confocal laser scanning microscope with META module (Carl Zeiss MicroImaging, Carl Zeiss Microscopy GmbH Strasse 22 73447 Oberkochen Deutschland) was used to detect the immunofluorescence, and Zen 2011 (LSM 700 Zeiss software) was employed to process the images [34,35,36]. Each image was rapidly acquired to minimize photodegradation. The sections treated with the anti-S100 protein after incubation were washed in the same buffer and incubated for 1.5 h at room temperature with secondary antibody-peroxidase conjugate (see Table 1). The immunoreaction was visualized using 3-30-diaminobenzidine as a chromogen (DAB, Sigma-Aldrich, Inc., St. Louis, MO, USA, cat. #D5905) [37]. After rinsing in freshwater, sections were dehydrated, mounted, and examined under Leica DMRB light microscope. Representative sections were incubated with appropriately preabsorbed antisera as mentioned above to provide negative controls. In these circumstances, there was no evidence of positive immunostaining.

### 2.3. Statistical Analysis

ImageJ software (Version 1.53t) was used to evaluate microscope fields collected randomly. One-way ANOVA was used to examine the statistical significance of the quantity of retinal pigment epithelium (RPE), photoreceptor layer (PRL), outer plexiform layer (OPL), amacrine cells (ACs), inner plexiform layer (IPL), bipolar cells (BCs), and ganglion cells (GCs) detected by Calretinin N-18, Parvalbumin and S100 protein. SigmaPlot version 14.0 (Systat Software, San Jose, CA, USA) was used to conduct the statistical analysis. An unpaired Z test was also performed. The information was given as median values with standard deviations (Δσ). Values of *p* below 0.05 were considered statistically significant in the following order *** *p* < 0.001, ** *p* < 0.01, * *p* < 0.05.

## 3. Results

**Figure 1:**
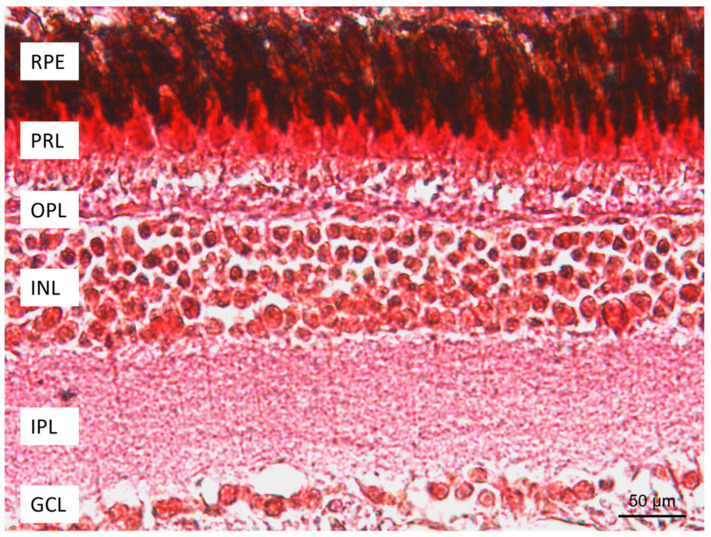
Retina of *N. guentheri*: RPE, retinal pigment epithelium; PRL, photoreceptor layer; OPL, outer plexiform layer; INL, inner nuclear layer; IPL, inner plexiform layer; GCL, ganglion cell layer; Masson trichrome with aniline blue method. Magnification 40×.

In order to analyze the localization of the calcium-binding proteins (CaBPs) Calretinin N-18, Parvalbumin, and S100, an immunohistochemistry study was conducted. The cells immunoreactive to CaBPs were identified by the topographic approach and using the specificity of anti-Calretinin N-18 and anti-Parvalbumin antibodies for retinal neurons. The morphological investigation of *N. guentheri* retina showed a similar organization to other vertebrates. The retina of *N. guentheri* was formed in seven layers: retinal pigment epithelium (RPE), photoreceptor layer (PRL) containing cones and rods, outer nuclear layer (ONL), inner nuclear layer (INL), ganglion cell layer (GCL), the outer plexiform layer (OPL) between ONL and INL, and the inner plexiform layer (IPL) between INL and GCL (Figure 1).

**Figure 2:**
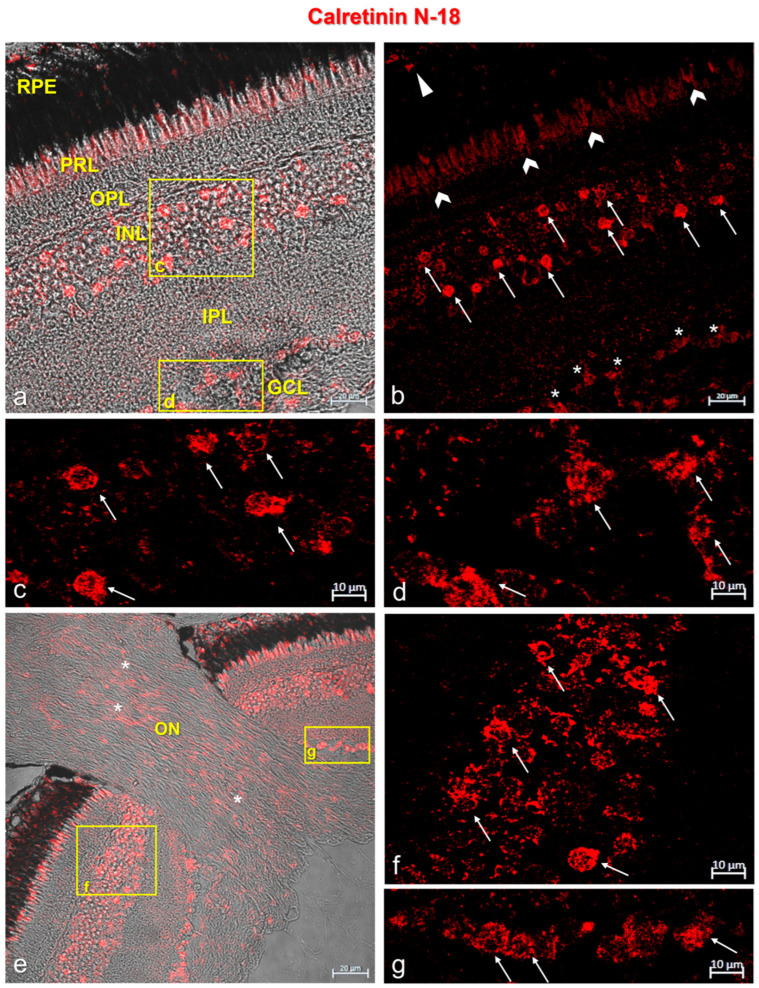
Calretinin N-18 immunostaining in *N. guentheri* retina. (**a**) Transmitted light of Calretinin N-18 immunostaining. This image shows the stratigraphy of the *N. guentheri* retina: RPE, retinal pigment epithelium; PRL, photoreceptor layer; OPL, outer plexiform layer; INL, inner nuclear layer; IPL, inner plexiform layer; GCL, ganglion cell layer. (**b**) Calretinin-N18 immunoreactivity in the cytoplasmic prolongations of the cells of the retinal pigment epithelium (RPE) (arrowhead); in the cones and rods of the photoreceptors layer (PRL) (chevron arrows); in a subpopulation of amacrine cells in the inner nuclear layer (INL) (arrows); in the soma of ganglion cells (GCL) (asterisk). (**c**) High magnification of the inset in (**a**) showing a Calretinin-N18-immunopositive subpopulation of amacrine cells (arrows) in the INL. (**d**) High magnification of the inset in (**a**) showing Calretinin-N18 immunostained soma of the ganglion cells (GCL) (arrows). (**e**) Transmitted light of Calretinin-N18 immunostaining of optic nerve (ON, asterisk) in *N. guentheri* retina. (**f**) High magnification of inset in (**e**) showing a subpopulation of Calretinin -N18-immunoreactive amacrine cells (arrows) in the INL. (**g**) High magnification of inset in (**e**) showing the soma of Calretinin-N18-immunostained ganglion cells (GCL) (arrows). Magnification: 40× (**a**,**b**,**e**), 63× (**c**,**d**,**f**,**g**).

**Figure 3:**
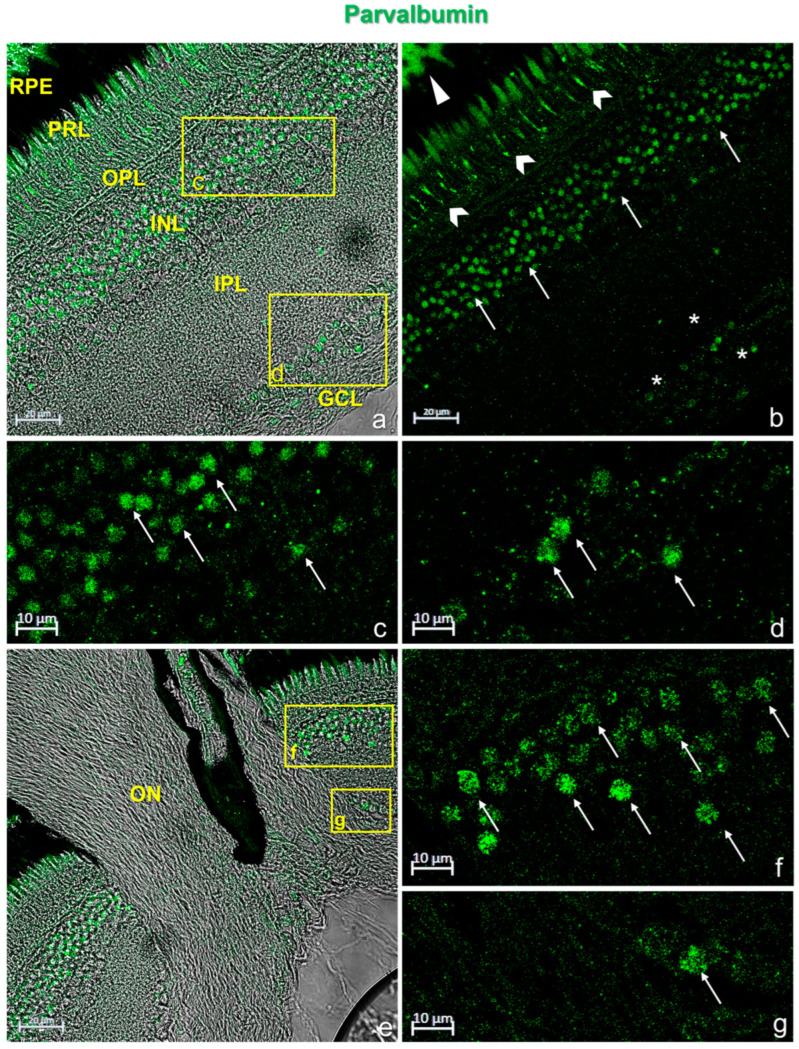
Parvalbumin immunostaining in *N. guentheri* retina. (**a**) Transmitted light of Parvalbumin immunostaining. Stratigraphy of the *N. guentheri* retina: RPE, retinal pigment epithelium; PRL, photoreceptor layer; OPL, outer plexiform layer; INL, inner nuclear layer; IPL, inner plexiform layer; GCL, ganglion cell layer. (**b**) Parvalbumin immunoreactivity in the cytoplasmic prolongations of the cells of the retinal pigment epithelium (RPE) (arrowhead); in the cones and rods of the photoreceptors layer (PRL) (chevron arrows); in a subpopulation of amacrine cells in the inner nuclear layer (INL) (arrows); in the soma of ganglion cells (GCL) (asterisk). (**c**) High magnification of the inset in (**a**) showing a subpopulation of Calretinin-N18-immunoreactive amacrine cells in the INL (arrows). (**d**) High magnification of the inset in (**a**) showing the soma of the Parvalbumin-immunostained ganglion cells (GCL) (arrows). (**e**) Transmitted light of Parvalbumin immunostaining in *N. guentheri* retina. No immunoreaction to Parvalbumin was found in the optic nerve (ON). (**f**) High magnification of inset in (**e**) showing a subpopulation of Parvalbumin-immunoreactive amacrine cells (arrows) in the INL. (**g**) High magnification of inset in (**e**) showing the soma of Parvalbumin-immunostained ganglion cells (GCL) (arrows). Magnification: 40× (**a**,**b**,**e**), 63× (**c**,**d**,**f**,**g**).

In the retina of *N. guentheri*, Calretinin N-18 and Parvalbumin were immunolocalized in the cytoplasmatic prolongation of the retinal pigment epithelium (RPE) and in the photoreceptor layer (PRL). A subpopulation of amacrine cells in the outer plexiform layer (OPL) and ganglion cells showed immunopositivity to Calretinin N-18 and Parvalbumin (Figure 2 and Figure 3). In addition, the optic nerve of *N. guentheri* showed Calretinin-N18 immunostained (Figure 2e) but not immunoreactivity to Parvalbumin (Figure 3e).

**Figure 4:**
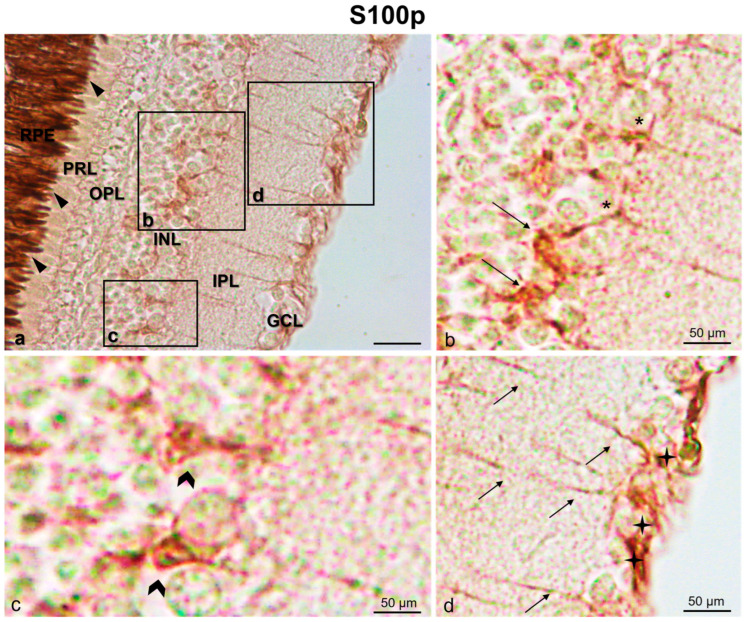
S100 protein immunostaining (indirect immunoperoxidase methos) in *N. guentheri* retina. (**a**) S100 immunoreactivity in the cytoplasmic prolongations of the cells in the retinal pigment epithelium (RPE) (arrowheads). (**b**) High magnification of inset in (**a**) showing a subpopulation of amacrine cells (arrows) and some horizontal cells (asterisks) immunoreative to S100p. (**c**) High magnification of inset in (**a**) showing S100-immunoreactive bipolar cells (gallon arrows). (**d**) High magnification of inset in (**a**) with S100-immunoreactive ganglion cells (stars) and axons in inner plexiform layer (arrows). Magnification: 20× (**a**), 40×, (**b**–**d**).

A subpopulation of amacrine cells and some bipolar and horizontal cells was immunopositive to S100p. Moreover, the ganglion cells were S100p immunostained. Additionally, the axons of amacrine cells, bipolar cells, and ganglion cells in the inner plexiform layer (IPL) were immunoreactive to S100p (Figure 4).

**Figure 5:**
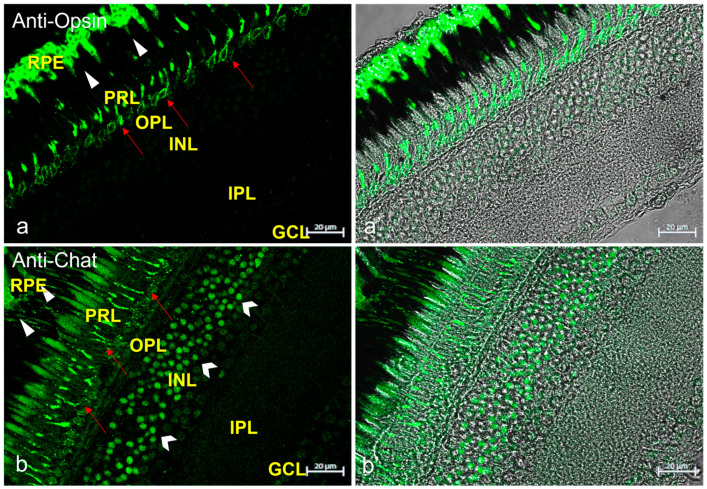
Anti-Opsin and anti-Chat immunostaining in *N. guentheri* retina. (**a**) Anti-Opsin immunoreactivity in the soma of rods (red arrows) and in the cytoplasmatic prolongation of retinal pigment epithelium (RPE) (arrowheads). (**a’**) transmitted light of anti-Opsin immunostaining. (**b**) Anti-Chat immunoreactivity in a subpopulation of amacrine cells (gallon arrows), in the prolongation of rods (red arrows), in the cytoplasmatic prolongation of the retinal pigment epithelium) (arrowheads). (**b’**) transmitted light of anti-Chat immunostaining. Magnification 40×.

To ascertain the cellular identity of the immunopositive cells shown, the immunoreaction of anti-Opsin (specific for rods) and anti-Chat (specific for amacrine cells) antibodies was investigated (Figure 5).

**Figure 6:**
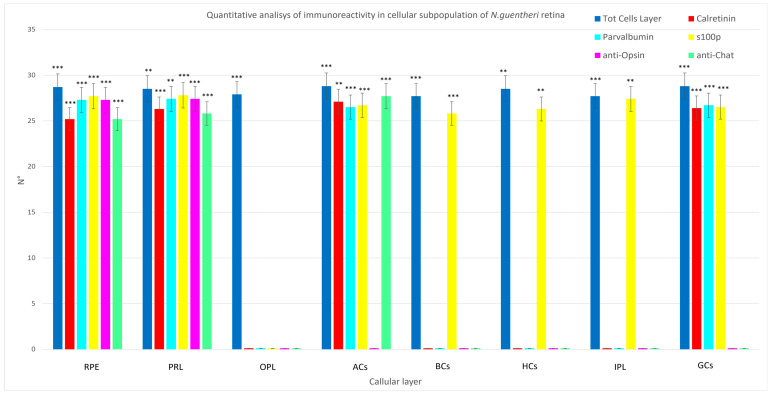
Graphical representation of immunoreactivity quantitative analysis in retinal pigment epithelium (RPE), photoreceptor layer (PRL), outer plexiform layer (OPL), amacrine cells (ACs), inner plexiform layer (IPL), bipolar cells (BCs), ganglion cells (GCs) detected by Calretinin N-18, Parvalbumin, S100 protein, anti-Opsin, and anti-Chat in comparison to the total cells of a layer. The statistical analysis shows a different distribution pattern of the antibodies used in this study in the cellular layer of *N. guentheri* retina. N°: mean of retinal layer cells immunoreactive to Calretinin-N18, Parvalbumin, and S100 protein. Statistical significance: *** *p* < 0.001, ** *p* < 0.01.

According to the results of quantitative analysis, Calretinin N-18, Parvalbumin, and S100 antibodies were immunolocalized in RPE, PRL, INL, and GCL. In particular, S100 p was found in different subpopulations of INL (amacrine cells, bipolar cells, horizontal cells), in GCL, and in IPL. The OPL did not show immunoreativity to Calretinin N-18, Parvalbumin, and S100p. A comparison of Calretinin N-18, Parvalbumin, and S100p in different cellular layers of *N. guentheri* retina is shown in Figure 6 and Table 2.

## 4. Discussion

In the 21st century, life expectancy has increased due to improved living conditions and medical advances. Contextually, the incidence of age-related disorders has risen too.

Aging is a significant risk factor both for non-neurodegenerative and neurodegenerative eye diseases. For instance, about 50 million people worldwide suffer from neurodegenerative diseases [38,39] affecting the visual system and the rest of the central nervous system, while on the other hand aging affects not only how well the visual system works but also how well it can safeguard and restore damaged or degenerating neurons [40,41].

The most recent WHO estimates place the number of people with visual impairments at 285 million. Unfortunately, there is no cure for the neurodegenerative eye condition due to aging or not. Moreover, in general, the study of aging is challenging because of the lack of an experimental model with a long life cycle.

However, killifish (*Nothobranchius* spp.) is a great aging model to fill this lack because it has a relatively short life cycle in comparison to other vertebrate models and numerous aging characteristics that have already been identified in humans [40,42]. In particular, it has been noted that Nothobranchius’ central nervous system exhibits peculiar signs of aging of all the vertebrates [43,44,45,46,47,48,49,50,51].

Further, *Nothobranchius* spp. appear to be suitable models to research age-dependent cellular and molecular processes and/or neurodegenerative events.

The visual system of *N. guentheri* and of other fish species is similar, as demonstrated by Dmitry et al. [52], and the anatomy of the retina is comparable to that of other vertebrates, such as humans. The retina of *N. guentheri* has stratigraphy that is comparable to that of vertebrates, such as humans and *Danio rerio* [53], and few differences from other teleosts mainly related to habitat, feeding, and reproduction. Shortly before the end of the life cycle of *N. guentheri*, the retinal layers weaken, and the epithelial layer of the pigment shrinks, as observed in *Oryzias latipes* and *D. rerio* [54,55] and other vertebrates including only humans [56,57]. Finally, investigations on the *N. guentheri* retina’s development have shown characteristics of neurogenesis and regeneration [52]. For these reasons, the retina of the annual killifish appears to be an ideal model in biomedical studies [52]. In both fish and mammals, the visual system is regarded as a crucial tool for understanding the brain as a whole. The central nervous system includes the retina as a necessary component [58]. Since the retina is seen as a window on the brain, recent research [59,60,61,62] has found that abnormal processes in the retina may reflect parallel processes in the central nervous system and vice versa. For instance, during Alzheimer’s disease (AD), specific pathological findings in the brain occur in the retina also [63]. As a matter of fact, it has been demonstrated that Aβ plaques appear earlier in the retina of AD animals than in the brain [64].

A common denominator in brain aging, in the pathogenesis of different neurodegenerative diseases, retinal pathology, and age-related degeneration of the retina is a disturbance in calcium balance and signaling [65,66].

In this context, CaBPs’ regulation may exert an influence on cellular survival. Calretinin N-18, Parvalbumin, S100p, and other members of the CaBPs’ family are involved in calcium balance control on which crucial cellular functions rely (e.g., gene expression, cell cycle progression, synaptic transmission, and apoptosis) [67,68]. In the peripheral and central nervous systems, the retina included, CaBPs are extensively disseminated [19,20,21,22] and they are employed as markers of specific nerve cells [69,70,71,72,73]. Specifically, Car-N18 is involved in calcium signaling regulation and neuronal excitability modulation [74,75,76]. Parvalbumin is related to the occurrence of various clinical diseases and age-related cognitive deficits and nervous system disorders [77]. The S100 protein is recognized as a marker of sensory cells and expressed in the nervous system of fish and other vertebrates [21,32,70,71,78,79,80]. It has already been found in several areas of the central nervous system of *N. furzeri* [81,82,83,84]. Urvashi and Shamsher [85] have shown the role of protein s 100 in neurodegenerative disease. Furthermore, calcium-binding proteins are of fundamental significance for the proper functioning of the neurotrophin/receptor system [70,86,87,88,89,90].

To our knowledge, there are no reports of CaBPs in the retina of the aging-emerging model *N. guentheri* in the current scientific literature. In this study, we show for the first time the localization of the calcium-binding proteins Calretinin N-18, Parvalbumin, and S100p in *N. guentheri* retina.

The occurrence in the retina of calcium-binding proteins has been studied extensively, but their neural function in retinal layers still remains unclear.

According to our data, Calretinin N-18 was present in the pigmented epithelium and in the photoreceptors layer of *N. guentheri*, such as in zebrafish and rats. On the other hand, our result are not compliant with the literature regarding Calretinin localization in the inner and outer plexiform layer of rats. In *N. guentheri* and in humans, the outer plexiform layer is not Calretinin N-18-immuoreactive, unlike rats in which the outer plexiform layer was Calretinin-immunopositive [73,91,92,93,94,95,96,97,98,99]. These data could corroborate the suitability of *N. guentheri* as a model for translational medicine.

Moreover, Calretinin N-18 was found in *N. guentheri* ganglion cell layer as in other model organisms (rats and mice) and humans.

*N. guentheri* showed Parvalbumin-immunopositivity in pigmented epithelium, but it has never been investigated in other model organisms and humans according to our knowledge. The photoreceptor layer of *N. guentheri* was Parvalbumin-immunoreactive as in zebrafish. Finally, Parvalbumin was localized in ganglion cell layer of *N. guentheri* as in other model organisms and humans [76,95,99,100,101,102,103,104,105].

Immunoreactivity to S100 was observed in pigmented epithelium of *N. guentheri* and humans. The ganglion cell layer of *N. guentheri* was immunopositive to S100p, as in rats, mice, and humans [73,93,94,96,104]. The inner nuclear layer of *N. guentheri* retina showed immunoreactivity to Calretinin and S100p as in rats, mice, and humans [73,91,92,93,94,96,99,100].

Calretinin, Parvalbumin, and S100p are distributed separately in the subpopulations of neurons in the nuclear layers of the retina [106,107], although species-specific variations exist. Regarding the inner nuclear layer, the results of our investigation on *N. guentheri* showed Calretinin N-18 and Parvalbumin localization in the amacrine cells. The S100p was immunoreactive amacrine, bipolar, and horizontal cells of *N. guentheri*. In the retina of rabbit, anti-Calretinin antibodies mark amacrine cells (AII cells), bipolar cells, and numerous cellular bodies in the ganglion cell layer [107,108,109], while anti-Parvalbumin antibodies stain amacrine cells (AII cells), horizontal cells, and ganglion cells [107,110]. Amacrine cells are the main cell type expressing Calretinin in vertebrates. Wässle et al. [111,112] identified the amacrine AII type cells and some rods in the retina of *M. fascicularis* as Calretinin-reactive. Comparable data were submitted by Bordt et al. [113] and Chiquet et al. [114]. Also, another subpopulation of amacrine cells and ganglion cells in the human retina is Calretinin-immunopositive [96]. Horizontal cells in the outer part of the inner plexiform layer and their processes in the outer plexiform layer are the primary type of Parvalbumin-positive cell in the retina of *Macaca* sp. and *Cercopithecus aethiops* [115,116,117]. Parvalbumin-positive amacrine and ganglion cells were found [118]. To compare the localization of CaBPs (Calretinin N-18, Parvalbumin, S100p) in different retinal cell layers of the different models with humans, see Table 3.

In agreement with the existing literature, our data show that each CaBPs is specifically expressed in subtypes of retinal neurons [118].

Finally, a consistent piece of evidence in the scientific literature reports the neuroprotective role of the CaBPs. CaBPs showed a protective role against toxicity caused by the increased release of neurotransmitters by regulating intracellular calcium levels [138,139] and that their expression can change during neurodegenerative conditions [140] and during pathological conditions affecting the retina [91,140,141,142]. Therefore, it could be speculated that the expression of CaBPs can be studied to understand the mechanisms of age-related damage of retina [104].

## 5. Conclusions

The present study showed the localization identification of calcium-binding proteins (CaBPs) Calretinin N-18, Parvalbumin, and S100p in *N. guentheri* retina for the first time. The localization of Calretinin N-18 and Parvalbumin in the retina of *N. guentheri* could demonstrate the neuroprotective role of these two CaBPs during aging and validate anti-Calretinin and anti-Parvalbumin antibodies as specific markers to identify subpopulations of retinal neurons to facilitate the study of retinal impairments induced by aging and/or neurodegenerative diseases. Future studies are needed to better understand the role of CaBPs and their expression patterns during the aging process and/or in transgenic specimens for neurodegenerative diseases.

## Figures and Tables

**Table 1:** Antibodies used in this study.

**Primary Antibodies**	**Supplier**	**Catalog Number**	**Source**	**Dilution**	**Antibody ID**
Calretinin-N18	Santa Cruz Biotechnology, Inc., Dallas, TX, USA	sc-11644	goat	1:100	AB_634545
Parvalbumin clone PA235	Sigma-Aldrich, Inc., St. Louis, MO, USA	P-3171	mouse	1:1000	AB_2313693
S100	Dako Agilent, Santa Clara, CA, USA	Z0311	rabbit	1:100	AB_10013383
Anti-Opsina Clone RET-P1	Sigma-Aldrich, Inc., St. Louis, MO, USA	O4886	mouse	1:100	AB_260838
Anti-Chat	Sigma-Aldrich, Inc., St. Louis, MO, USA	AMAB91130	mouse	1:100	AB_2665812
**Secondary Antibodies**	**Supplier**	**Catalog Number**	**Source**	**Dilution**	**Antibody ID**
Antigoat IgG (H + L) Alexa Fluor 594	Molecular Probes, Invitrogen, Waltham, MA, USA	A-11058	donkey	1:300	AB_2534105
Antimouse IgG (H + L) Alexa Fluor 488	Molecular Probes, Invitrogen, Waltham, MA, USA	A-11001	goat	1:300	AB_2534069
Antirabbit IgG peroxidase conjugate	Amersham Bioscences, Amersham, United Kingdom	NA934	donkey	1:100	AB_772206

**Table 2:** Mean data ± standard deviation (∆σ) of immunopositivity of: GC (ganglion cell); IPL (inner plexiform layer); INL (inner nuclear layer); OPL (outer plexiform layer); ONL (outer nuclear layer); RPE (retinal pigment epithelium) detected by Calretinin N-18, Parvalbumin, S100 protein, anti-Opsin, and anti-Chat in comparison to the total cells of a layer. The statistical analysis shows a different distribution pattern of the antibodies used in this study in cellular layer of *N. guentheri* retina. All features were evaluated per 174.286 ± 3.082 µm (mean). Statistical significance: *** *p* < 0.001, ** *p* < 0.01.

	Total Cells of a Layer	Calretinin N-18	Parvalbumin	S100	Anti-Opsin	Anti-Chat
**Mean ± ∆σ in RPE**	28.7 ± 4.9 ***	25.2 ± 4.5 ***	27.3 ± 3.57 ***	27.7 ± 5.36 ***	27.3 ± 3.57 ***	25.2 ± 4.5 ***
**Mean ± ∆σ in PRL**	28.5 ± 4.7 **	26.3 ± 3.1 ***	27.4 ± 4.8 **	27.8 ± 5.97 ***	27.4 ± 4.8 **	28.5 ± 4.7 **
**Mean ± ∆σ in OPL**	27.9 ± 5.6 ***	n/a	n/a	n/a	n/a	n/a
**Mean ± ∆σ in ACs**	28.8 ± 5.11 ***	27.1 ± 4.08 **	26.5 ± 5.48 ***	26.7 ± 4.64 ***	n/a	27.7 ± 5.36 ***
**Mean ± ∆σ in BCs**	27.7 ± 5.36 ***	n/a	n/a	25.8 ± 4.6 ***	n/a	n/a
**Mean ± ∆σ in HCs**	28.5 ± 4.7 **	n/a	n/a	26.3 ± 4 **	n/a	n/a
**Mean ± ∆σ in IPL**	27.7 ± 5.36 ***	n/a	n/a	27.4 ± 4.84 ***	n/a	n/a
**Mean ± ∆σ in GCs**	28.8 ± 5.11 ***	26.4 ± 5.40 ***	26.7 ± 4.42 ***	26.5 ± 5.12 ***	n/a	n/a

**Table 3:** Comparison of different species’ CaBPs (Calretinin N-18, Parvalbumin, S100p) with regard to the localization and expression in the retina layers of *N. guentheri*.

Species	*Nothobranchius guentheri* *			Zebrafish			Ref	Rat			Ref	Mouse			Ref	Human			Ref
Antibodies	Calretinin N-18	Parvalbumin	S100p	Calretini N-18	Parvalbumin	S100p		Calretinin N-18	Parvalbumin	S100p		Calretini N-18	Parvalbumin	S100p		Calretini N-18	Parvalbumin	S100p	
**RPE**	+	+	+	+	n/a	n/a	[91]	+	n/a	n/a	[92]	n/a	n/a	n/a		n/a	n/a	+	[93,94]
**PRL**	+	+	−	+	+	n/a	[119]	+	n/a	n/a	[92,95]	n/a	n/a	n/a	[120]	−	n/a	n/a	[76,95,101,121,122]
**OPL**	−	−	−	−	n/a	n/a	[119]	+	n/a	+	[95,123,124]	n/a	+	n/a	[100,125]	−	n/a	+	[93,94,95,126]
**INL**	+	+	+	n/a	+	n/a	[119]	+	n/a	+	[92,95,103,110,123,127,128,129]	+	n/a		[98,99]	+	n/a	+	[73,96,130]
**Bipolar Cells (INL)**	−	−	+	−	n/a	n/a	[119]	−	n/a	n/a	[92,131]	−	n/a		[76]	+	+	+	[76,94,95,96]
**Amacrine Cells (INL)**	+	+	+	−	n/a	n/a		+	+	n/a	[95,101,103,104,105,132,133]	+	+		[76,100,134]	+	+	n/a	[76,95,96,101]
**IPL**	−	−	+	−	+	n/a	[119]	+	+	n/a	[92,95,99,103,123,128]	+	+	n/a	[98,99,100,134,135]	+	n/a	n/a	[93,96,136]
**GLC**	+	+	+	−	+	n/a	[119]	+	+	+	[92,95,101,104,105,110,123,127,128,137]	+	+	n/a	[76,99,100,134]	+	+	+	[76,94,95,96,101,122,130]

(*) these data refer to the sample of the present study. Retinal pigment epithelium (RPE), photoreceptor layer (PRL), outer plexiform layer (OPL), inner plexiform layer (INL), inner plexiform layer (IPL), ganglion cell layer (GCL). (+) positive for the considered antibody; (−) negative for the considered antibody; (n/a) references data not known, to the best of our knowledge.

## Data Availability

All data presented this study are available from the corresponding author, upon responsible request.

## References

[1] Purves D., Augustine G., Fitzpatrick D., Hall W., LaMantia A., McNamara E., White J.O. (2008). Neuroscience.

[2] Schoch S., Gundelfinger E.D. (2006). Molecular organization of the presynaptic active zone. Cell Tissue Res..

[3] Südhof T.C. (2004). The Synaptic Vesicle Cycle. Annu. Rev. Neurosci..

[4] Zhai R.G., Bellen H.J. (2004). The Architecture of the Active Zone in the Presynaptic Nerve Terminal. Physiology.

[5] Dresbach T., Qualmann B., Kessels M.M., Garner C.C., Gundelfinger E.D. (2001). The presynaptic cytomatrix of brain synapses. Cell. Mol. Life Sci. CMLS.

[6] Südhof T.C. (2013). Neurotransmitter release: The last millisecond in the life of a synaptic vesicle. Neuron.

[7] Boczek T., Mackiewicz J., Sobolczyk M., Wawrzyniak J., Lisek M., Ferenc B., Guo F., Zylinska L. (2021). The Role of G Protein-Coupled Receptors (GPCRs) and Calcium Signaling in Schizophrenia. Focus on GPCRs Activated by Neurotransmitters and Chemokines. Cells.

[8] Dhyani V., Gare S., Gupta R.K., Swain S., Venkatesh K.V., Giri L. (2020). GPCR mediated control of calcium dynamics: A systems perspective. Cell Signal..

[9] Roberts W.M. (1994). Localization of calcium signals by a mobile calcium buffer in frog saccular hair cells. J. Neurosci..

[10] Neher E. (1988). The influence of intracellular calcium concentration on degranulation of dialysed mast cells from rat peritoneum. J. Physiol..

[11] Stern M.D. (1992). Buffering of calcium in the vicinity of a channel pore. Cell Calcium.

[12] Castro A., Becerra M., Anadón R., Manso M.J. (2008). Distribution of calretinin during development of the olfactory system in the brown trout, *Salmo trutta* fario: Comparison with other immunohistochemical markers. J. Chem. Neuroanat..

[13] Castro A., Becerra M., Manso M.J., Anadón R. (2006). Calretinin immunoreactivity in the brain of the zebrafish, Danio rerio: Distribution and comparison with some neuropeptides and neurotransmitter-synthesizing enzymes. I. Olfactory organ and forebrain. J. Comp. Neurol..

[14] Germanà A., Paruta S., Germanà G.P., Ochoa-Erena F.J., Montalbano G., Cobo J., Vega J.A. (2007). Differential distribution of S100 protein and calretinin in mechanosensory and chemosensory cells of adult zebrafish (*Danio rerio*). Brain Res..

[15] Levanti M.B., Montalbano G., Laurà R., Ciriaco E., Cobo T., García-Suarez O., Germanà A., Vega J. (2008). Calretinin in the peripheral nervous system of the adult zebrafish. J. Anat..

[16] Münkle M.C., Waldvogel H.J., Faull R.L. (1999). Calcium-binding protein immunoreactivity delineates the intralaminar nuclei of the thalamus in the human brain. Neuroscience.

[17] Yan K., Tang Y.Z., Carr C.E. (2010). Calcium-binding protein immunoreactivity characterizes the auditory system of Gekko gecko. J. Comp. Neurol..

[18] Fairless R., Williams S.K., Diem R. (2019). Calcium-Binding Proteins as Determinants of Central Nervous System Neuronal Vulnerability to Disease. Int. J. Mol. Sci..

[19] Langeh U., Singh S. (2021). Targeting S100B Protein as a Surrogate Biomarker and its Role in Various Neurological Disorders. Curr. Neuropharmacol..

[20] Landfield P.W. (1987). ‘Increased calcium-current’ hypothesis of brain aging. Neurobiol. Aging.

[21] Khachaturian Z.S. (1994). Calcium Hypothesis of Alzheimer’s Disease and Brain Aginga. Ann. N. Y. Acad. Sci..

[22] Fiuza F.P., Queiroz J.P.G., Aquino A.C.Q., Câmara D.A., Brandão L.E.M., Lima R.H., Cavalcanti J.R.L., Engelberth R.C.G., Cavalcante J.S. (2021). Aging Alters Daily and Regional Calretinin Neuronal Expression in the Rat Non-image Forming Visual Thalamus. Front. Aging Neurosci..

[23] Lamerand S., Shahidehpour R., Ayala I., Gefen T., Mesulam M.M., Bigio E., Geula C. (2020). Calbindin-D28K, parvalbumin, and calretinin in young and aged human locus coeruleus. Neurobiol. Aging.

[24] Wang X., Chang Q., Wang Y., Su F., Zhang S. (2014). Late-onset temperature reduction can retard the aging process in aged fish via a combined action of an anti-oxidant system and the insulin/insulin-like growth factor 1 signaling pathway. Rejuvenation Res..

[25] Wang X., Du X., Zhou Y., Wang S., Su F., Zhang S. (2017). Intermittent food restriction initiated late in life prolongs lifespan and retards the onset of age-related markers in the annual fish *Nothobranchius guentheri*. Biogerontology.

[26] Wang X., Ren Y., Du X., Song L., Chen F., Su F. (2020). Effects of late-onset dietary intake of salidroside on insulin/insulin-like growth factor-1 (IGF-1) signaling pathway of the annual fish *Nothobranchius guentheri*. Arch. Gerontol. Geriatr..

[27] Li C., Song L., Zhou Y., Yuan J., Zhang S. (2022). Identification of Isthmin1 in the small annual fish, *Nothobranchius guentheri*, as a novel biomarker of aging and its potential rejuvenation activity. Biogerontology.

[28] Matsunaga H., Handa J.T., Aotaki-Keen A., Sherwood S.W., West M.D., Hjelmeland L.M. (1999). Beta-galactosidase histochemistry and telomere loss in senescent retinal pigment epithelial cells. Investig. Ophthalmol. Vis. Sci..

[29] Liu C., Wang X., Feng W., Li G., Su F., Zhang S. (2012). Differential expression of aging biomarkers at different life stages of the annual fish *Nothobranchius guentheri*. Biogerontology.

[30] Yildirim Z., Ucgun N.I., Yildirim F. (2011). The role of oxidative stress and antioxidants in the pathogenesis of age-related macular degeneration. J. Clin..

[31] Aragona M., Porcino C., Guerrera M.C., Montalbano G., Levanti M., Abbate F., Laurà R., Germanà A. (2021). Localization of Neurotrophin Specific Trk Receptors in Mechanosensory Systems of Killifish (*Nothobranchius guentheri*). Int. J. Mol. Sci..

[32] Abbate F., Guerrera M.C., Montalbano G., De Carlos F., Suárez A.Á., Ciriaco E., Germanà A. (2012). Morphology of the european sea bass (*Dicentrarchus labrax*) tongue. Microsc. Res. Tech..

[33] Lauriano E., Guerrera M., Laurà R., Capillo G., Pergolizzi S., Aragona M., Abbate F., Germanà A. (2020). Effect of light on the calretinin and calbindin expression in skin club cells of adult zebrafish. Histochem. Cell Biol..

[34] Lauriano E.R., Capillo G., Icardo J.M., Fernandes J.M.O., Kiron V., Kuciel M., Zuwala K., Guerrera M.C., Aragona M., Germana’ A. (2021). Neuroepithelial cells (NECs) and mucous cells express a variety of neurotransmitters and neurotransmitter receptors in the gill and respiratory air-sac of the catfish *Heteropneustes fossilis* (Siluriformes, Heteropneustidae): A possible role in local immune defence. Zoology.

[35] Capillo G., Zaccone G., Cupello C., Fernandes J.M.O., Viswanath K., Kuciel M., Zuwala K., Guerrera M.C., Aragona M., Icardo J.M. (2021). Expression of acetylcholine, its contribution to regulation of immune function and O_2_ sensing and phylogenetic interpretations of the African butterfly fish *Pantodon buchholzi* (Osteoglossiformes, Pantodontidae). Fish Shellfish Immunol..

[36] Licata P., Tardugno R., Pergolizzi S., Capillo G., Aragona M., Colombo A., Gervasi T., Pellizzeri V., Cicero N., Calò M. (2018). In vivo effects of PCB-126 and genistein on vitellogenin expression in zebrafish. Nat. Prod. Res..

[37] United Nations (2020). World Population Ageing 2019 Division.

[38] Cao W., Li T. (2020). COVID-19: Towards understanding of pathogenesis. Cell Res..

[39] Vanhunsel S., Beckers A., Moons L. (2020). Designing neuroreparative strategies using aged regenerating animal models. Ageing Res. Rev..

[40] Chader G.J., Taylor A. (2013). Preface: The Aging Eye: Normal Changes, Age-Related Diseases, and Sight-Saving Approaches. Investig. Ophthalmol. Vis. Sci..

[41] López-Otín C., Blasco M.A., Partridge L., Serrano M., Kroemer G. (2013). The Hallmarks of Aging. Cell.

[42] Genade T., Benedetti M., Terzibasi E., Roncaglia P., Valenzano D.R., Cattaneo A., Cellerino A. (2005). Annual fishes of the genus Nothobranchius as a model system for aging research. Aging Cell.

[43] Terzibasi E., Valenzano D.R., Cellerino A. (2007). The short-lived fish *Nothobranchius furzeri* as a new model system for aging studies. Exp. Gerontol..

[44] Valenzano D.R., Cellerino A. (2006). Resveratrol and the Pharmacology of Aging: A New Vertebrate Model to Validate an Old Molecule. Cell Cycle.

[45] Valenzano D.R., Sharp S., Brunet A. (2011). Transposon-mediated transgenesis in the short-lived African killifish *Nothobranchius furzeri*, a vertebrate model for aging. G3 Genes Genomes Genet..

[46] Tozzini E.T., Baumgart M., Battistoni G., Cellerino A. (2012). Adult neurogenesis in the short-lived teleost Nothobranchius furzeri: Localization of neurogenic niches, molecular characterization and effects of aging. Aging Cell.

[47] Tozzini E.T., Cellerino A. (2020). Nothobranchius annual killifishes. EvoDevo.

[48] Baumgart M., Groth M., Priebe S., Savino A., Testa G., Dix A., Ripa R., Spallotta F., Gaetano C., Ori M. (2014). RNA-seq of the aging brain in the short-lived fish *N. furzeri*–conserved pathways and novel genes associated with neurogenesis. Aging Cell.

[49] Hartmann N., Englert C. (2012). A microinjection protocol for the generation of transgenic killifish (Species: *Nothobranchius furzeri*). Dev. Dyn..

[50] Hartmann N., Reichwald K., Wittig I., Dröse S., Schmeisser S., Lück C., Hahn C., Graf M., Gausmann U., Terzibasi E. (2011). Mitochondrial DNA copy number and function decrease with age in the short-lived fish *Nothobranchius furzeri*. Aging Cell.

[51] Nikiforov-Nikishin D.L., Irkha V.A., Kochetkov N.I., Kalita T.L., Nikiforov-Nikishin A.L., Blokhin E.E., Antipov S.S., Makarenkov D.A., Zhavnerov A.N., Glebova I.A. (2021). Some Aspects of Development and Histological Structure of the Visual System of Nothobranchius Guentheri. Animals.

[52] Tarboush R., Chapman G.B., Connaughton V.P. (2012). Ultrastructure of the distal retina of the adult zebrafish, *Danio rerio*. Tissue Cell.

[53] Menke A.L., Spitsbergen J.M., Wolterbeek A.P., Woutersen R.A. (2011). Normal anatomy and histology of the adult zebrafish. Toxicol. Pathol..

[54] Kishi S., Slack B.E., Uchiyama J., Zhdanova I.V. (2009). Zebrafish as a Genetic Model in Biological and Behavioral Gerontology: Where Development Meets Aging in Vertebrates—A Mini-Review. Gerontology.

[55] Hanus J., Anderson C., Wang S. (2015). RPE necroptosis in response to oxidative stress and in AMD. Ageing Res. Rev..

[56] Fabre M., Mateo L., Lamaa D., Baillif S., Pagès G., Demange L., Ronco C., Benhida R. (2022). Recent Advances in Age-Related Macular Degeneration Therapies. Molecules.

[57] Bollaerts I., Veys L., Geeraerts E., Andries L., De Groef L., Buyens T., Salinas-Navarro M., Moons L., Van Hove I. (2018). Complementary research models and methods to study axonal regeneration in the vertebrate retinofugal system. Brain Struct. Funct..

[58] Mirzaei N., Shi H., Oviatt M., Doustar J., Rentsendorj A., Fuchs D.-T., Sheyn J., Black K.L., Koronyo Y., Koronyo-Hamaoui M. (2020). Alzheimer’s retinopathy: Seeing disease in the eyes. Front. Neurosci..

[59] Guo L., Normando E.M., Shah P.A., De Groef L., Cordeiro M.F. (2018). Oculo-visual abnormalities in Parkinson’s disease: Possible value as biomarkers. Mov. Disord..

[60] Vandenabeele M., Veys L., Lemmens S., Hadoux X., Gelders G., Masin L., Serneels L., Theunis J., Saito T., Saido T.C. (2021). The AppNL-G-F mouse retina is a site for preclinical Alzheimer’s disease diagnosis and research. Acta Neuropathol. Commun..

[61] Veys L., Vandenabeele M., Ortuño-Lizarán I., Baekelandt V., Cuenca N., Moons L., De Groef L. (2019). Retinal α-synuclein deposits in Parkinson’s disease patients and animal models. Acta Neuropathol..

[62] Parnell M., Guo L., Abdi M., Cordeiro M.F. (2012). Ocular Manifestations of Alzheimer’s Disease in Animal Models. Int. J. Alzheimer’s Dis..

[63] Koronyo-Hamaoui M., Koronyo Y., Ljubimov A.V., Miller C.A., Ko M.K., Black K.L., Schwartz M., Farkas D.L. (2011). Identification of amyloid plaques in retinas from Alzheimer’s patients and noninvasive in vivo optical imaging of retinal plaques in a mouse model. NeuroImage.

[64] Cortes L., Malva J., Rego A.C., Pereira C.F. (2020). Calcium Signaling in Aging and Neurodegenerative Diseases 2019. Int. J. Mol. Sci..

[65] Schmidt K.-G., Bergert H., Funk R. (2008). Neurodegenerative diseases of the retina and potential for protection and recovery. Curr. Neuropharmacol..

[66] Andressen C., Blümcke I., Celio M.R. (1993). Calcium-binding proteins: Selective markers of nerve cells. Cell Tissue Res..

[67] Schwaller B. (2012). The use of transgenic mouse models to reveal the functions of Ca^2+^ buffer proteins in excitable cells. Biochim. Biophys. Acta (BBA)-Gen. Subj..

[68] Chin D., Means A.R. (2000). Calmodulin: A prototypical calcium sensor. Trends Cell Biol..

[69] Germana A., Abbate F., González-Martínez T., Del Valle M., De Carlos F., Germanà G., Vega J. (2004). S100 protein is a useful and specific marker for hair cells of the lateral line system in postembryonic zebrafish. Neurosci. Lett..

[70] Germanà A., Marino F., Guerrera M.C., Campo S., de Girolamo P., Montalbano G., Germanà G.P., Ochoa-Erena F.J., Ciriaco E., Vega J.A. (2008). Expression and distribution of S100 protein in the nervous system of the adult zebrafish *(Danio rerio*). Microsc. Res. Tech..

[71] Parisi V., Guerrera M.C., Abbate F., Garcia-Suarez O., Viña E., Vega J.A., Germanà A. (2014). Immunohistochemical characterization of the crypt neurons in the olfactory epithelium of adult zebrafish. Ann. Anat.-Anat. Anz..

[72] Kántor O., Mezey S., Adeghate J., Naumann A., Nitschke R., Énzsöly A., Szabó A., Lukáts Á., Németh J., Somogyvári Z. (2016). Calcium buffer proteins are specific markers of human retinal neurons. Cell Tissue Res..

[73] Airaksinen M.S., Thoenen H., Meyer M. (1997). Vulnerability of Midbrain Dopaminergic Neurons in Calbindin-D28k-deficient Mice: Lack of Evidence for a Neuroprotective Role of Endogenous Calbindin in MPTPtreated and Weaver Mice. Eur. J. Neurosci..

[74] Camp A.J., Wijesinghe R. (2009). Calretinin: Modulator of neuronal excitability. Int. J. Biochem. Cell Biol..

[75] Kovács-Öller T., Szarka G., Ganczer A., Tengölics Á., Balogh B., Völgyi B. (2019). Expression of Ca^2+^-Binding Buffer Proteins in the Human and Mouse Retinal Neurons. Int. J. Mol. Sci..

[76] Verret L., Mann E.O., Hang G.B., Barth A.M.I., Cobos I., Ho K., Devidze N., Masliah E., Kreitzer A.C., Mody I. (2012). Inhibitory Interneuron Deficit Links Altered Network Activity and Cognitive Dysfunction in Alzheimer Model. Cell.

[77] Germanà A., Guerrera M.C., Laurà R., Levanti M., Aragona M., Mhalhel K., Germanà G., Montalbano G., Abbate F. (2020). Expression and Localization of BDNF/TrkB System in the Zebrafish Inner Ear. Int. J. Mol. Sci..

[78] Abbate F., Catania S., Germana A., González T., Diaz-Esnal B., Germana G., Vega J. (2002). S-100 protein is a selective marker for sensory hair cells of the lateral line system in teleosts. Neurosci. Lett..

[79] Abbate F., Guerrera M.C., Montalbano G., Ciriaco E., Germanà A. (2012). Morphology of the tongue dorsal surface of gilthead seabream (*Sparus aurata*). Microsc. Res. Tech..

[80] D’angelo L. (2013). Brain Atlas of an Emerging Teleostean Model: *Nothobranchius furzeri*. Anat. Rec..

[81] D’Angelo L., de Girolamo P., Cellerino A., Tozzini E.T., Castaldo L., Lucini C. (2012). Neurotrophin Trk receptors in the brain of a teleost fish, *Nothobranchius furzeri*. Microsc. Res. Tech..

[82] D’Angelo L., De Girolamo P., Lucini C., Terzibasi E.T., Baumgart M., Castaldo L., Cellerino A. (2014). Brain-derived neurotrophic factor: mRNA expression and protein distribution in the brain of the teleost *Nothobranchius furzeri*. J. Comp. Neurol..

[83] Leggieri A., Attanasio C., Palladino A., Cellerino A., Lucini C., Paolucci M., Terzibasi Tozzini E., de Girolamo P., D’Angelo L. (2019). Identification and expression of neurotrophin-6 in the brain of *Nothobranchius furzeri*: One more piece in neurotrophin research. J. Clin. Med..

[84] Aragona M., Porcino C., Guerrera M.C., Montalbano G., Laurà R., Cometa M., Levanti M., Abbate F., Cobo T., Capitelli G. (2022). The BDNF/TrkB Neurotrophin System in the Sensory Organs of Zebrafish. Int. J. Mol. Sci..

[85] Aragona M., Porcino C., Guerrera M.C., Montalbano G., Laurà R., Levanti M., Abbate F., Cobo T., Capitelli G., Calapai F. (2022). Localization of BDNF and Calretinin in Olfactory Epithelium and Taste Buds of Zebrafish (*Danio rerio*). Int. J. Mol. Sci..

[86] Copray J.C.V.M., Mantingh-Otter I.J., Brouwer N. (1994). Expression of calcium-binding proteins in the neurotrophin-3-dependent subpopulation of rat embryonic dorsal root ganglion cells in culture. Dev. Brain Res..

[87] Miguel J.C., Perez S.E., Malek-Ahmadi M., Mufson E.J. (2021). Cerebellar Calcium-Binding Protein and Neurotrophin Receptor Defects in Down Syndrome and Alzheimer’s Disease. Front. Aging Neurosci..

[88] Germana A., Montalbano G., Laura R., Ciriaco E., Del Valle M., Vega J.A. (2004). S100 protein-like immunoreactivity in the crypt olfactory neurons of the adult zebrafish. Neurosci. Lett..

[89] Porcino C., Briglia M., Aragona M., Mhalhel K., Laurà R., Levanti M., Abbate F., Montalbano G., Germanà G., Lauriano E.R. (2023). Potential Neuroprotective Role of Calretinin-N18 and Calbindin-D28k in the Retina of Adult Zebrafish Exposed to Different Wavelength Lights. Int. J. Mol. Sci..

[90] Hwang I.K., Yoo K.Y., Kim D.S., Jung J.Y., Shin M.C., Seo K., Kim K.S., Kang T.C., Won M.H. (2005). Comparative Study on Calretinin Immunoreactivity in Gerbil and Rat Retina. Anat. Histol. Embryol..

[91] Nag T.C., Wadhwa S. (1996). Calbindin and parvalbumin immunoreactivity in the developing and adult human retina. Dev. Brain Res..

[92] Nag T.C., Wadhwa S. (1999). Developmental expression of calretinin immunoreactivity in the human retina and a comparison with two other EF-hand calcium-binding proteins. Neuroscience.

[93] Hamano K., Kiyama H., Emson P.C., Manabe R., Nakauchi M., Tohyama M. (1990). Localization of two calcium binding proteins, calbindin (28 kD) and parvalbumin (12 kD), in the vertebrate retina. J. Comp. Neurol..

[94] Lee S.C.S., Weltzien F., Madigan M.C., Martin P.R., Grünert U. (2016). Identification of AII amacrine, displaced amacrine, and bistratified ganglion cell types in human retina with antibodies against calretinin. J. Comp. Neurol..

[95] Takahashi-Iwanaga H., Shimoda H. (2003). The three-dimensional microanatomy of Meissner corpuscles in monkey palmar skin. J. Neurocytol..

[96] Fu Z., Nian S., Li S.-Y., Wong D., Chung S.K., Lo A.C.Y. (2015). Deficiency of aldose reductase attenuates inner retinal neuronal changes in a mouse model of retinopathy of prematurity. Graefe’s Arch. Clin. Exp. Ophthalmol..

[97] Gábriel R., Erdélyi F., Szabó G., Lawrence J.J., Wilhelm M. (2016). Ectopic transgene expression in the retina of four transgenic mouse lines. Brain Struct. Funct..

[98] Haverkamp S., Wässle H. (2000). Immunocytochemical analysis of the mouse retina. J. Comp. Neurol..

[99] Celio M.R. (1990). Calbindin D-28k and parvalbumin in the rat nervous system. Neuroscience.

[100] Kovács-Öller T., Debertin G., Balogh M., Ganczer A., Orbán J., Nyitrai M., Balogh L., Kántor O., Völgyi B. (2017). Connexin36 expression in the mammalian retina: A multiple-species comparison. Front. Cell. Neurosci..

[101] Kovács-Öller T., Raics K., Orbán J., Nyitrai M., Völgyi B. (2014). Developmental changes in the expression level of connexin36 in the rat retina. Cell Tissue Res..

[102] Kovács-Valasek A., Pöstyéni E., Dénes V., Mester A., Sétáló Jr G., Gábriel R. (2021). Age-Related Alterations of Proteins in Albino Wistar Rat Retina. Cells Tissues Organs.

[103] Trost A., Schroedl F., Marschallinger J., Rivera F.J., Bogner B., Runge C., Couillard-Despres S., Aigner L., Reitsamer H.A. (2014). Characterization of dsRed2-positive cells in the doublecortin-dsRed2 transgenic adult rat retina. Histochem. Cell Biol..

[104] Massey S.C., Mills S.L. (1996). A calbindin-immunoreactive cone bipolar cell type in the rabbit retina. J. Comp. Neurol..

[105] Kwon O.-J., Kim J.-Y., Kim S.-Y., Jeon C.-J. (2005). Alterations in the localization of calbindin D28K-, calretinin-, and parvalbumin-immunoreactive neurons of rabbit retinal ganglion cell layer from ischemia and reperfusion. Mol. Cells.

[106] Jeon M.-H., Jeon C.-J. (1998). Immunocytochemical localization of calretinin containing neurons in retina from rabbit, cat, and dog. Neurosci. Res..

[107] Chang-Jin J., Enrica S., Richard H.M. (1998). The Major Cell Populations of the Mouse Retina. J. Neurosci..

[108] Casini G., Rickman D.W., Brecha N.C. (1995). AII amacrine cell population in the rabbit retina: Identification by parvalbumin immunoreactivity. J. Comp. Neurol..

[109] Wässle H., Peichl L., Airaksinen M.S., Meyer M. (1998). Calcium-binding proteins in the retina of a calbindin-null mutant mouse. Cell Tissue Res..

[110] Chun M.-H., Kim I.-B., Ju W.-K., Kim K.-Y., Lee M.-Y., Joo C.-K., Chung J.-W. (1999). Horizontal cells of the rat retina are resistant to degenerative processes induced by ischemia-reperfusion. Neurosci. Lett..

[111] Bordt A.S., Hoshi H., Yamada E.S., Perryman-Stout W.C., Marshak D.W. (2006). Synaptic input to OFF parasol ganglion cells in macaque retina. J. Comp. Neurol..

[112] Chiquet C., Dkhissi-Benyahya O., Cooper H.M. (2005). Calcium-binding protein distribution in the retina of strepsirhine and haplorhine primates. Brain Res. Bull..

[113] Röhrenbeck J., Wässle H., Boycott B.B. (1989). Horizontal Cells in the Monkey Retina: Immunocytochemical staining with antibodies against calcium binding proteins. Eur. J. Neurosci..

[114] Röhrenbeck J., Wässle H., Heizmann C.W. (1987). Immunocytochemical labelling of horizontal cells in mammalian retina using antibodies against calcium-binding proteins. Neurosci. Lett..

[115] Martin P.R., Grünert U. (1992). Spatial density and immunoreactivity of bipolar cells in the macaque monkey retina. J. Comp. Neurol..

[116] Roski C., Langrock C., Körber N., Habermann G., Buse E., Reichenbach A., Pannicke T., Francke M. (2018). Comparison of cellular localisation of the Ca^2+^-binding proteins calbindin, calretinin and parvalbumin in the retina of four different Macaca species. Anat. Histol. Embryol..

[117] Youn H.-Y., Chou B.R., Cullen A.P., Sivak J.G. (2009). Effects of 400nm, 420nm, and 435.8nm radiations on cultured human retinal pigment epithelial cells. J. Photochem. Photobiol. B Biol..

[118] Pochet R., Pasteels B., Seto-ohshima A., Bastianelli E., Kitajima S., Van Eldik L.J. (1991). Calmodulin and calbindin localization in retina from six vertebrate species. J. Comp. Neurol..

[119] Haley T.L., Pochet R., Baizer L., Burton M.D., Crabb J.W., Parmentier M., Polans A.S. (1995). Calbindin D-28K immunoreactivity of human cone cells varies with retinal position. Vis. Neurosci..

[120] Zhang C., Wang J., Zhou A., Ye Q., Feng Y., Wang Z., Wang S., Xu G., Zou J. (2021). Species-specific effect of microplastics on fish embryos and observation of toxicity kinetics in larvae. J. Hazard. Mater..

[121] Pöstyéni E., Szabadfi K., Sétáló G., Gabriel R. (2021). A Promising Combination: PACAP and PARP Inhibitor Have Therapeutic Potential in Models of Diabetic and Hypertensive Retinopathies. Cells.

[122] Kim S.A., Jung C.K., Kang T.-H., Jeon J.H., Cha J., Kim I.-B., Chun M.-H. (2010). Synaptic connections of calbindin-immunoreactive cone bipolar cells in the inner plexiform layer of rabbit retina. Cell Tissue Res..

[123] Sharma R.K., O’Leary T.E., Fields C.M., Johnson D.A. (2003). Development of the outer retina in the mouse. Dev. Brain Res..

[124] Zhang R., Zhang X., Hu F., Wu J. (2021). Fine structure of the human retina defined by confocal microscopic immunohistochemistry. Br. J. Biomed. Sci..

[125] Endo T., Kobayashi M., Kobayashi S., Onaya T. (1986). Immunocytochemical and biochemical localization of parvalbumin in the retina. Cell Tissue Res..

[126] Nivison-Smith L., Khoo P., Acosta M.L., Kalloniatis M. (2017). Pre-treatment with vinpocetine protects against retinal ischemia. Exp. Eye Res..

[127] Kim D., Kim M.J., Lee J.H., Im J.O., Won Y.J., Yoon S.-Y., Hong H.N. (2003). Concomitant distribution shift of glial GABA transporter and S100 calcium-binding proteins in the rat retina after kainate-induced excitotoxic injury. Neurosci. Lett..

[128] Iwanaga T., Takahashi Y., Fujita T. (1985). Immunohistochemical localization of S-100 protein in the retina, ciliary body and iris of human fetuses. Cell Tissue Res..

[129] Uesugi R., Yamada M., Mizuguchi M., Baimbridge K.G., Kim S.U. (1992). Calbindin D-28k and parvalbumin immunohistochemistry in developing rat retina. Exp. Eye Res..

[130] Wäussle H., Grüunert U., Röhrenbeck J. (1993). Immunocytochemical staining of AII-amacrine cells in the rat retina with antibodies against parvalbumin. J. Comp. Neurol..

[131] Oguni M., Setogawa T., Shinohara H., Kato K., Semba R. (1997). Distribution of γ-Aminobutyric Acid (GABA) and the Calcium-binding Protein Parvalbumin in Rat Retina during Development. Acta Histochem. Cytochem..

[132] Ho T., Vessey K.A., Fletcher E.L. (2014). Immunolocalization of the P2X4 receptor on neurons and glia in the mammalian retina. Neuroscience.

[133] Pasteels B., Rogers J., Blachier F., Pochet R. (1990). Calbindin and calretinin localization in retina from different species. Vis. Neurosci..

[134] Zhang W., Li C., Guo M. (2021). Use of ecofriendly alternatives for the control of bacterial infection in aquaculture of sea cucumber *Apostichopus japonicus*. Aquaculture.

[135] Sanna P.P., Keyser K.T., Battenberg E., Bloom F.E. (1990). Parvalbumin immunoreactivity in the rat retina. Neurosci. Lett..

[136] Miller R.J. (1995). Regulation of calcium homoeostasis in neurons: The role of calcium-binding proteins. Biochem. Soc. Trans..

[137] Lohmann C., Friauf E. (1996). Distribution of the calcium-binding proteins parvalbumin and calretinin in the auditory brainstem of adult and developing rats. J. Comp. Neurol..

[138] Kim I.-J., Zhang Y., Meister M., Sanes J.R. (2010). Laminar Restriction of Retinal Ganglion Cell Dendrites and Axons: Subtype-Specific Developmental Patterns Revealed with Transgenic Markers. J. Neurosci..

[139] Park H.-S., Park S.-J., Park S.-H., Chun M.-H., Oh S.-J. (2008). Shifting of parvalbumin expression in the rat retina in experimentally induced diabetes. Acta Neuropathol..

[140] Hernandez M., Rodriguez F.D., Sharma S.C., Vecino E. (2009). Immunohistochemical changes in rat retinas at various time periods of elevated intraocular pressure. Mol. Vis..

[141] Gunn D.J., Gole G.A., Barnett N.L. (2011). Specific amacrine cell changes in an induced mouse model of glaucoma. Clin. Exp. Ophthalmol..

[142] Huang J.-F., Shang L., Zhang M.-Q., Wang H., Chen D., Tong J.-B., Huang H., Yan X.-X., Zeng L.-P., Xiong K. (2013). Differential neuronal expression of receptor interacting protein 3 in rat retina: Involvement in ischemic stress response. BMC Neurosci..

